# Supplementary material for: Increased circulating levels of Factor H-Related Protein 4 are strongly associated with age-related macular degeneration
Source: Nat Commun. 2020 Feb 7;11:778. doi: 10.1038/s41467-020-14499-3 (PMC7005798; doi:10.1038/s41467-020-14499-3)
Supplement: Supplementary file 1 — Supplementary Information [file 41467_2020_14499_MOESM1_ESM.pdf]

SUPPLEMENTARY INFORMATION

Increased circulating levels of Factor H-Related Protein 4 are strongly associated with age-related macular degeneration

Cipriani *et al.*

Table of Contents

Supplementary Methods 3

Study samples 3

Supplementary Figure 1. Two-stage, fixed-effects meta-analysis of individual participant data from Cambridge and EUGENDA studies shows significant association of FHR-4 levels and late AMD. 4

Supplementary Figure 2. *CFHR4* gene transcription was not detected in eye tissues. 5

Supplementary Figure 3. FHR-4 does not diffuse freely across Bruch's membrane. 6

Supplementary Figure 4. FHL-1 mediated C3b breakdown assay. 7

Supplementary Figure 5. Box plots (and corresponding data points) of FHR-4 and FH levels measured in Cambridge and EUGENDA samples, by AMD status and genotype of 8 independently associated variants at the *CFH* locus from the IAMDGC study.[9] 8

Supplementary Figure 6. GWAS meta-analysis of FHR-4 levels in controls reveals a strong genome-wide significant signal spanning the *CFH* locus. 12

Supplementary Figure 7. Association analyses of the common diplotypes (haplotype pairs, with overall frequency ≥ 1%) formed by the 7 AMD independently associated variants at the *CFH* locus considered in our study and rs6677604 (proxy for the previously reported AMD protective *CFHR1-3* deletion[11]) with AMD, FHR-4 and FH levels. 14

Supplementary Figure 8. FHR-4 and FH levels are dictated by a different genetic architecture. 16

Supplementary Figure 9. Sequence of FHR-4 recombinant protein. 17

Supplementary Figure 10. Specificity of anti-FHR-4 antibody. 18

Supplementary Figure 11. Competition ELISA demonstrating specificity of anti-FHR4 antibody clone 150 for FHR-4 over FH. 20

Supplementary Note 1 21

List of the International Age-related Macular Degeneration Genomics Consortium (IAMDGC) members 21

Supplementary References 26

Supplementary Methods

Study samples

Cambridge AMD study patients were excluded if they had greater than 6 diopters of myopic refractive error or evidence of other inflammatory or retinovascular disease (such as retinal vessel occlusion, diabetic retinopathy, or chorioretinitis) that could contribute to the development of or confound the diagnosis of maculopathy. All participants described their race/ethnicity as white on a recruitment questionnaire and were confirmed to be of European descent in the genetic analyses. Participants were examined by an ophthalmologist and underwent color stereoscopic fundus photography of the macular region. Images were graded at the Reading Centre, Moorfields Eye Hospital, London, using the International Classification of Age-related Maculopathy and Macular Degeneration.[1]

For the European Genetic Database (EUGENDA) cohort, all the individuals were graded by classification of retinal images according to the standard protocol of the Cologne Image Reading Center by certified graders.[2] Only patients graded as late AMD were included in the study. Serum was obtained by a standard coagulation/centrifugation protocol, and within 1 hour after collection serum samples were stored at –80°C.

A

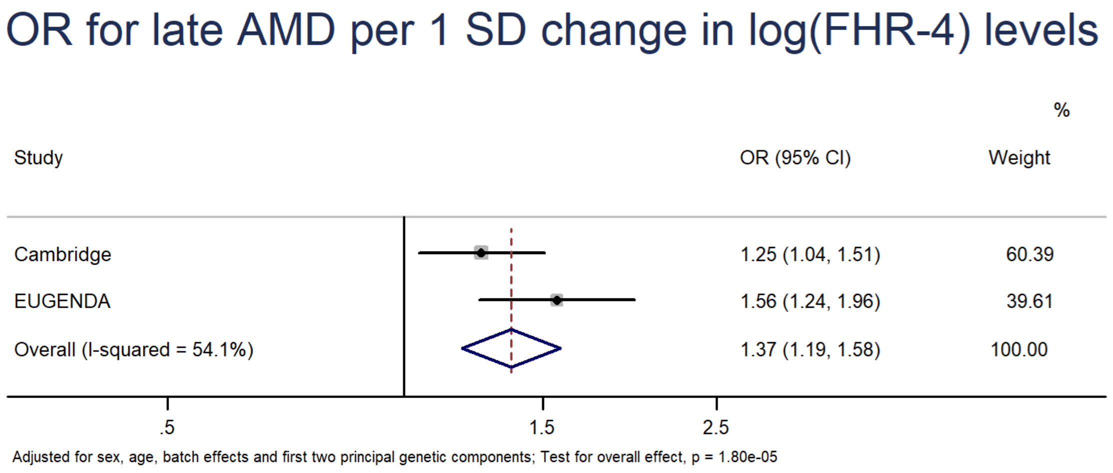

B

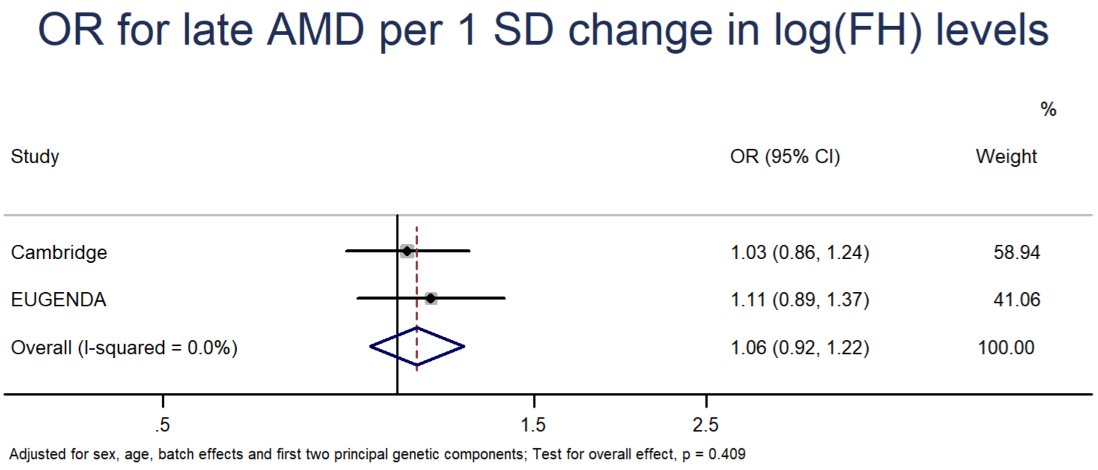

Supplementary Figure 1. Two-stage, fixed-effects meta-analysis of individual participant data from Cambridge and EUGENDA studies shows significant association of FHR-4 levels and late AMD.

Panels A and B show forest plots of odds ratios (ORs) (with 95% Confidence Intervals, CIs) of late AMD per standard deviation (SD) change in natural logarithmically transformed FHR-4 (A) and FH (B) levels using logistic regression models adjusted for sex, age, batch effects and the first two genetic principal components. The overall OR estimate is obtained from a two-stage, fixed-effects meta-analysis of the two study-specific estimates. I^2 statistic is used to assess heterogeneity across studies.

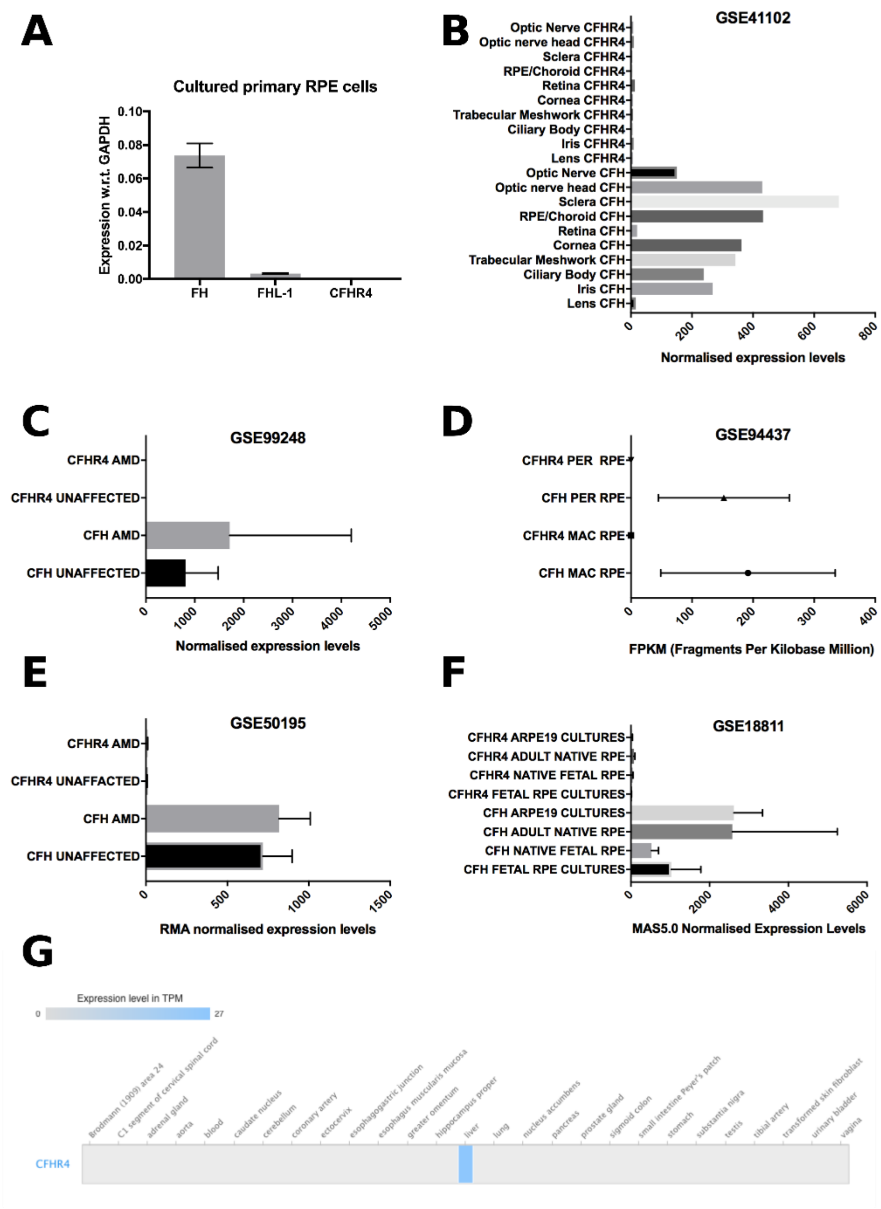

Supplementary Figure 2. *CFHR4* gene transcription was not detected in eye tissues. rtPCR analysis on cultured primary human RPE cells from 42 individual donors detects expression of FH and FHL-1, but not FHR-4 (panel A). Panels B-F show data reanalyzed from the NCBI Gene Expression Omnibus public data repository: where B is from an Affymetrix Human Exon 1.0 ST microarray[3]; C, RNAseq (Illumina)[4]; D, RNAseq (Illumina) HiSeq 2000[5]; E, Affymetrix Human Exon 1.0 ST microarray[6]; and F, Affymetrix U133plus2 human genome array[7]. Panel G: RNAseq of 53 human tissue samples from the Genotype-Tissue Expression (GTEx) project[8] detects *CFHR4* expression only in the liver. Error bars in panels A-F represent standard deviation. Source data are provided as a Source Data file.

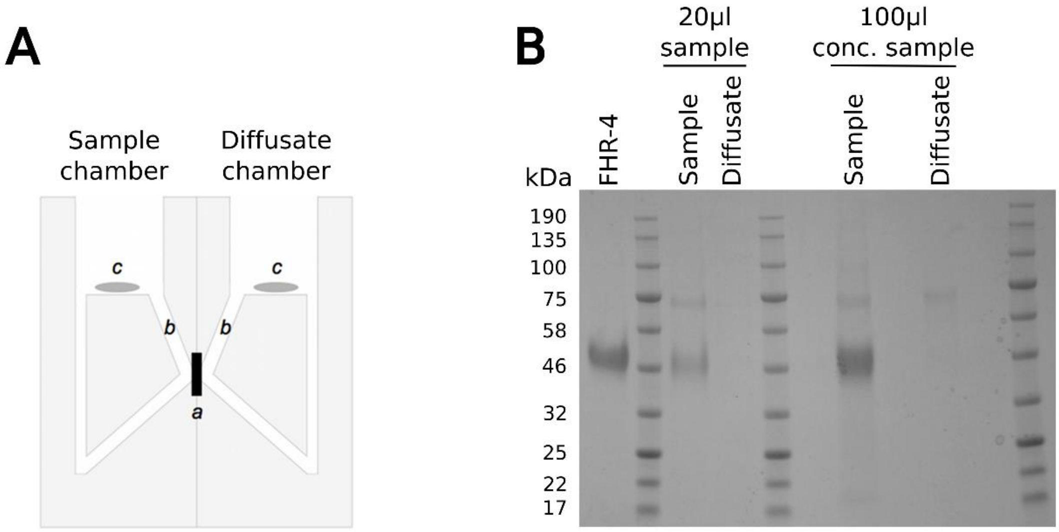

Supplementary Figure 3. FHR-4 does not diffuse freely across Bruch's membrane. Enriched Bruch's membrane from donor eyes were placed inside a modified Ussing chamber, where: a, is the enriched BrM; b, are the sampling access points; and c, are magnetic stirrer bars to maintain flow around each chamber (panel A). Panel B: samples from either the sample chamber or diffusate chamber were run on a 4-12% NuPage gel Bis-Tris gel and compared to a pure protein control (FHR-4); the protein in the gel was stained with Instant Blue. The gel shows 20µl samples taken and run directly from each chamber, as well as 100µl samples that have been concentrated prior to running on the gel. Gel is representative of three independent experiments.

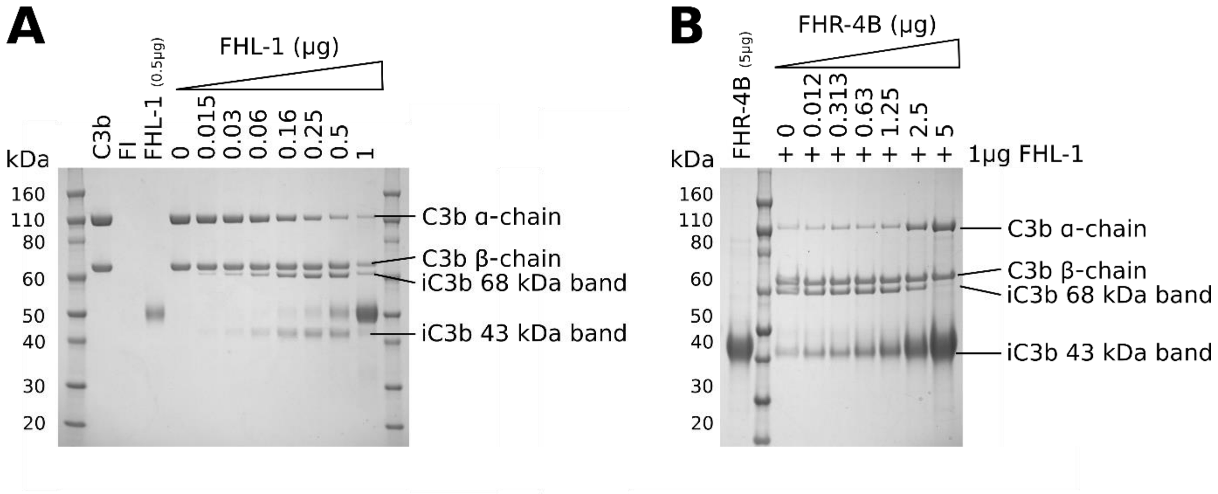

Supplementary Figure 4. FHL-1 mediated C3b breakdown assay.

Panel A: protein stained SDS-PAGE gel demonstrating FI cleavage of C3b in the fluid phase in the presence of a co-factor (FHL-1) is shown, with pure C3b (2µg), FI (0.04µg), and FHL-1 (0.5µg) controls included. FI cannot cleave the α-chain of C3b without a co-factor (lane '0'), but with increasing concentration of FHL-1 the breakdown of the C3b α-chain into iC3b (seen as two bands at 68kDa and 43kDa) was observed. Gel is representative of three independent experiments. Panel B: a repeat of the C3b breakdown assay as shown previously (panel A) but the amount of FHL-1 remains a constant 1µg and increasing amounts of FHR-4B purified protein is supplemented into the reaction. The 43kDa iC3b band is masked by the presence of FHR-4B. This competition assay gel is representative of three independent experiments.

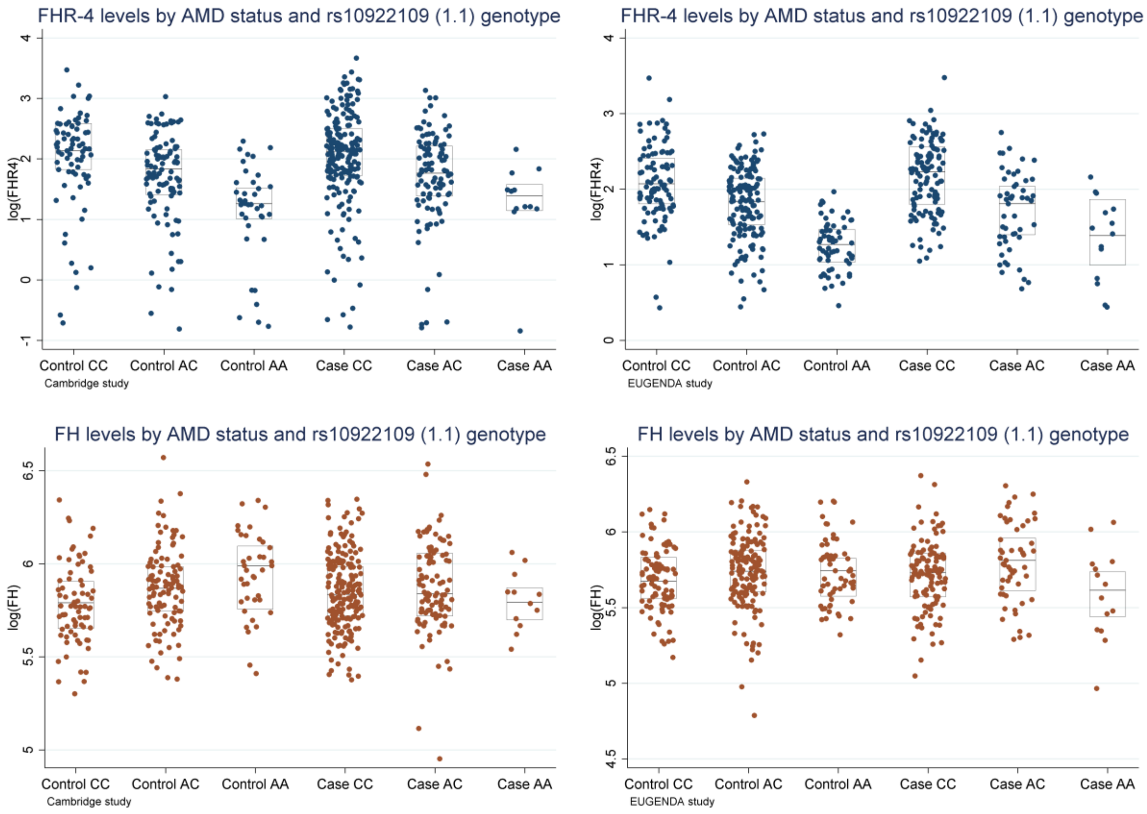

Supplementary Figure 5. Box plots (and corresponding data points) of FHR-4 and FH levels measured in Cambridge and EUGENDA samples, by AMD status and genotype of 8 independently associated variants at the *CFH* locus from the IAMDGC study.[9]

Each box plot depicts median value (central line), first quartile (lower bound line) and third quartile (upper bound line). Source data are provided as a Source Data file.

Note: the *CFH* variant rs121913059 (R1210C,[10] IAMDGC association signal number 1.3) was present heterozygously only in a single case from the Cambridge cohort and no corresponding box plot of FHR-4/FH levels is shown.

(continued on the next page)

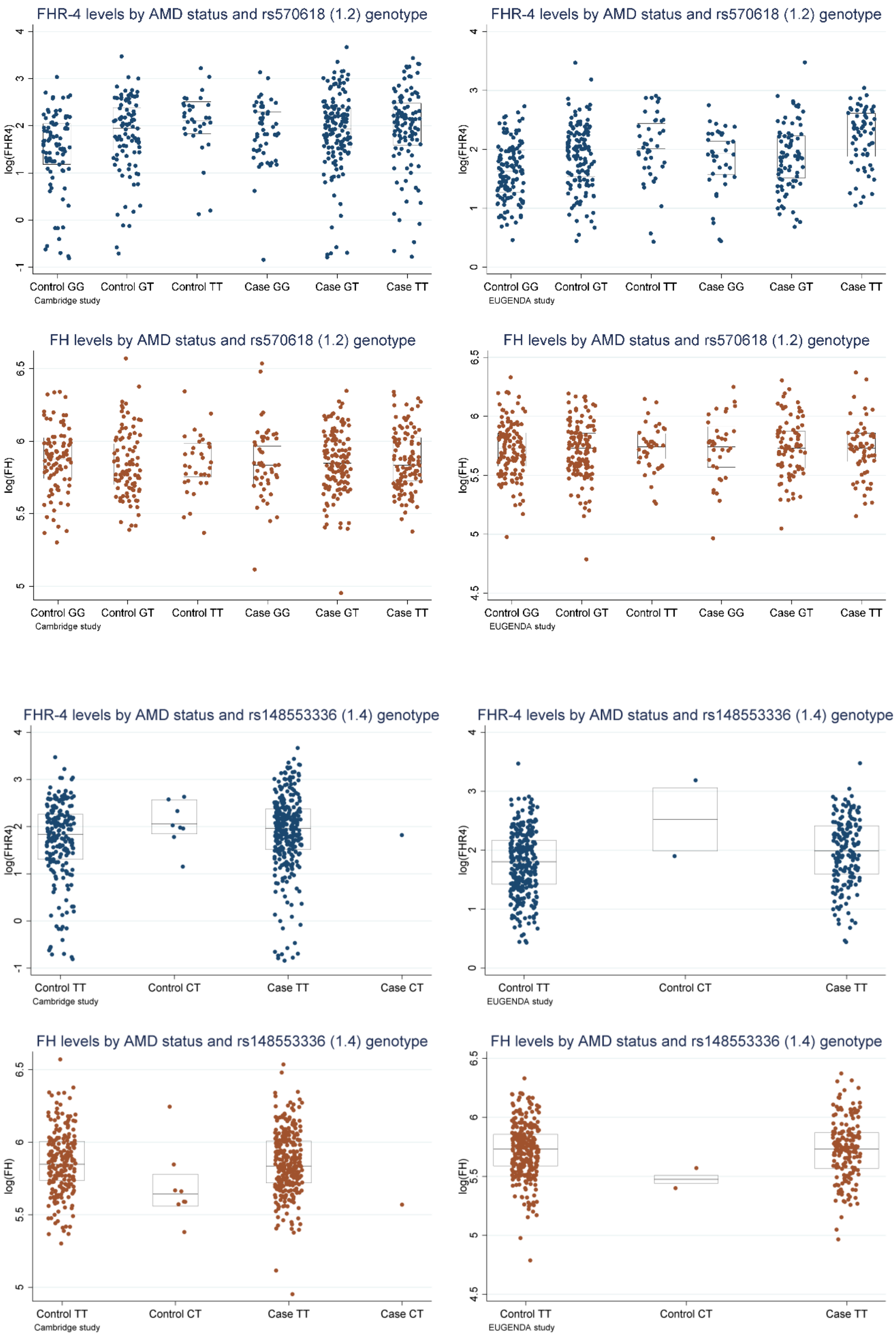

(continued on the next page)

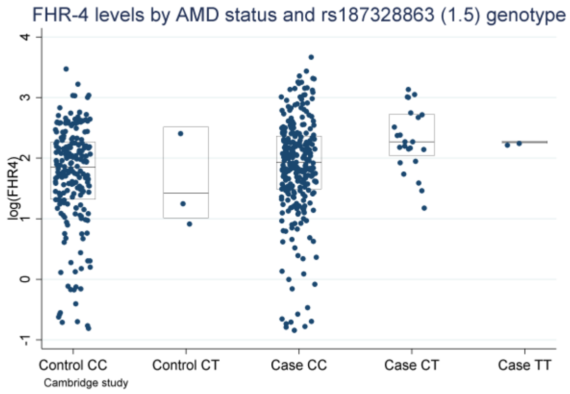

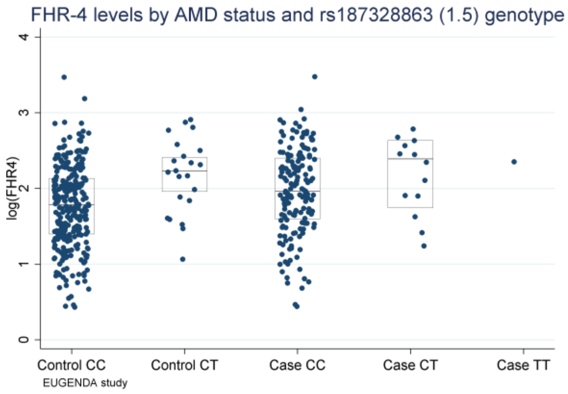

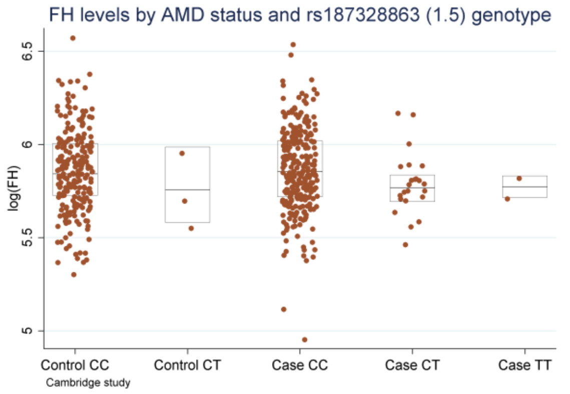

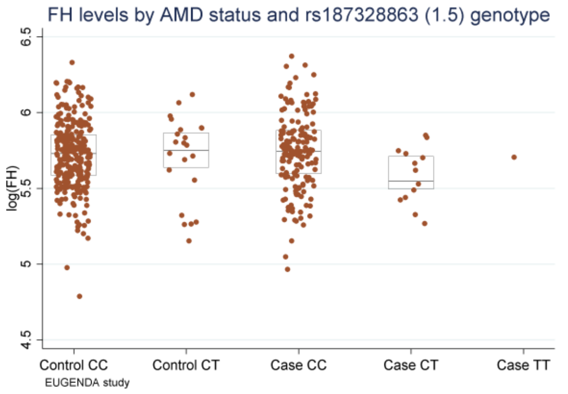

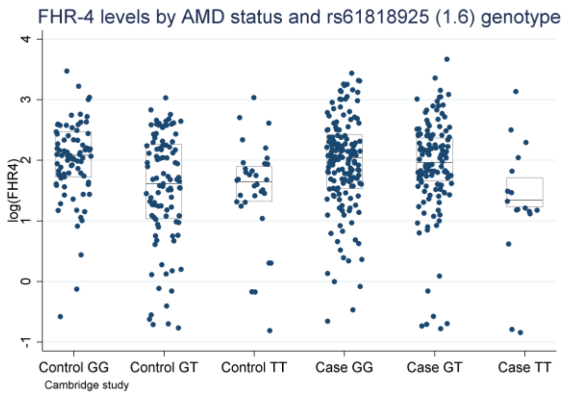

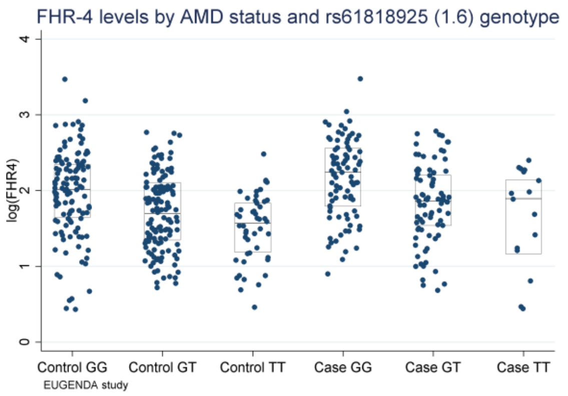

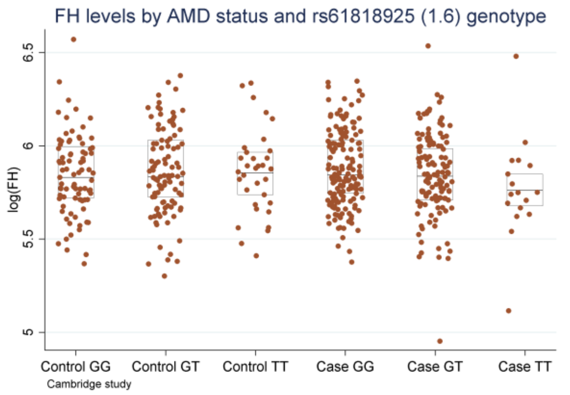

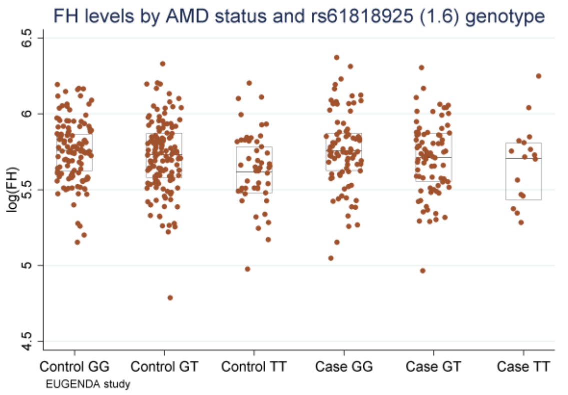

(continued on the next page)

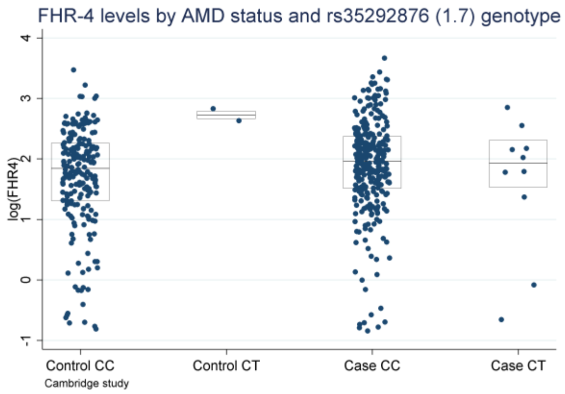
FHR-4 levels by AMD status and rs35292876 (1.7) genotype
log(FHR4)
-1
0
1
2
3
4
Control CC
Control CT
Case CC
Case CT
Cambridge study

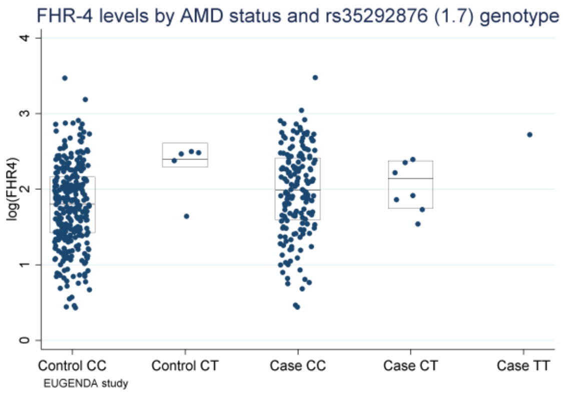
FHR-4 levels by AMD status and rs35292876 (1.7) genotype
log(FHR4)
0
1
2
3
4
Control CC
Control CT
Case CC
Case CT
Case TT
EUGENDA study

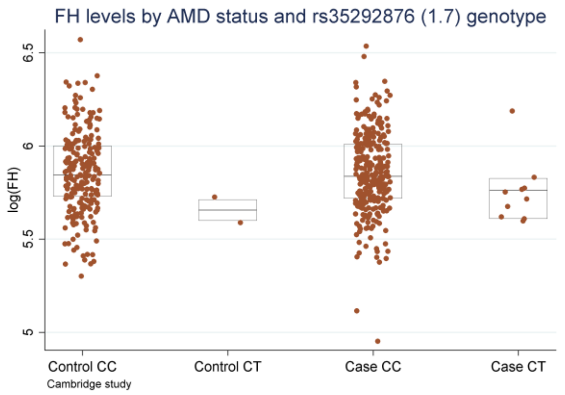
FH levels by AMD status and rs35292876 (1.7) genotype
log(FH)
5
5.5
6
6.5
Control CC
Control CT
Case CC
Case CT
Cambridge study

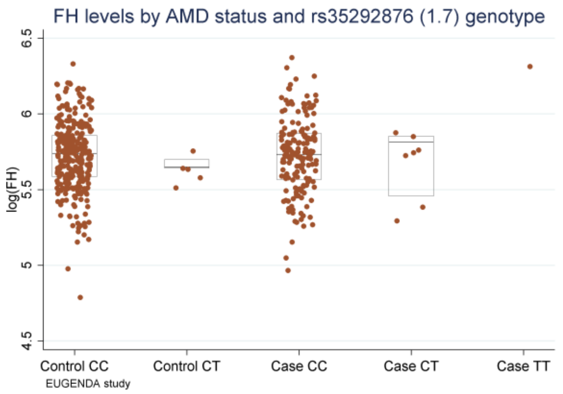
FH levels by AMD status and rs35292876 (1.7) genotype
log(FH)
4.5
5
5.5
6
6.5
Control CC
Control CT
Case CC
Case CT
Case TT
EUGENDA study

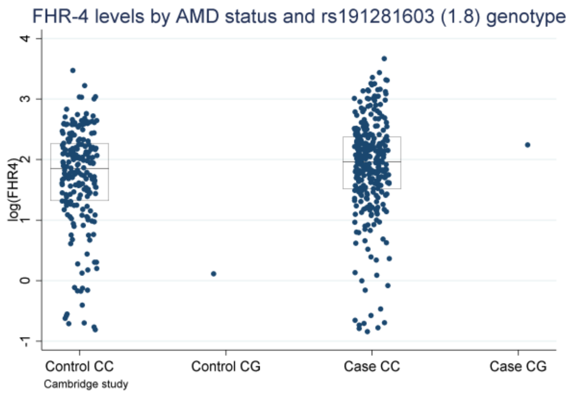
FHR-4 levels by AMD status and rs191281603 (1.8) genotype
log(FHR4)
-1
0
1
2
3
4
Control CC
Control CG
Case CC
Case CG
Cambridge study

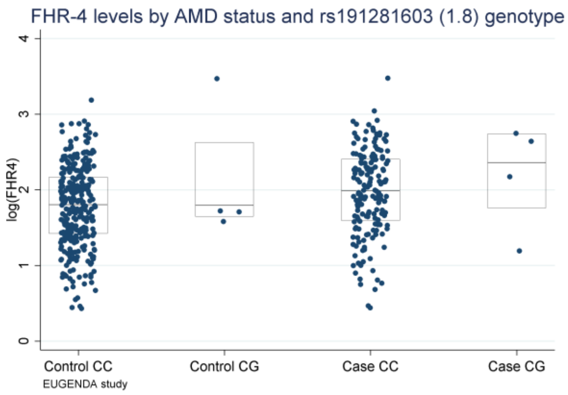
FHR-4 levels by AMD status and rs191281603 (1.8) genotype
log(FHR4)
0
1
2
3
4
Control CC
Control CG
Case CC
Case CG
EUGENDA study

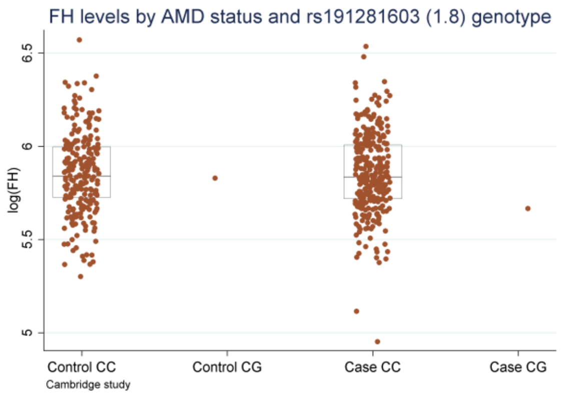
FH levels by AMD status and rs191281603 (1.8) genotype
log(FH)
5
5.5
6
6.5
Control CC
Control CG
Case CC
Case CG
Cambridge study

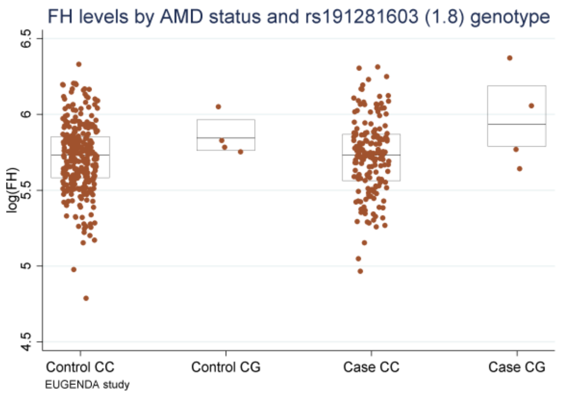
FH levels by AMD status and rs191281603 (1.8) genotype
log(FH)
4.5
5
5.5
6
6.5
Control CC
Control CG
Case CC
Case CG
EUGENDA study

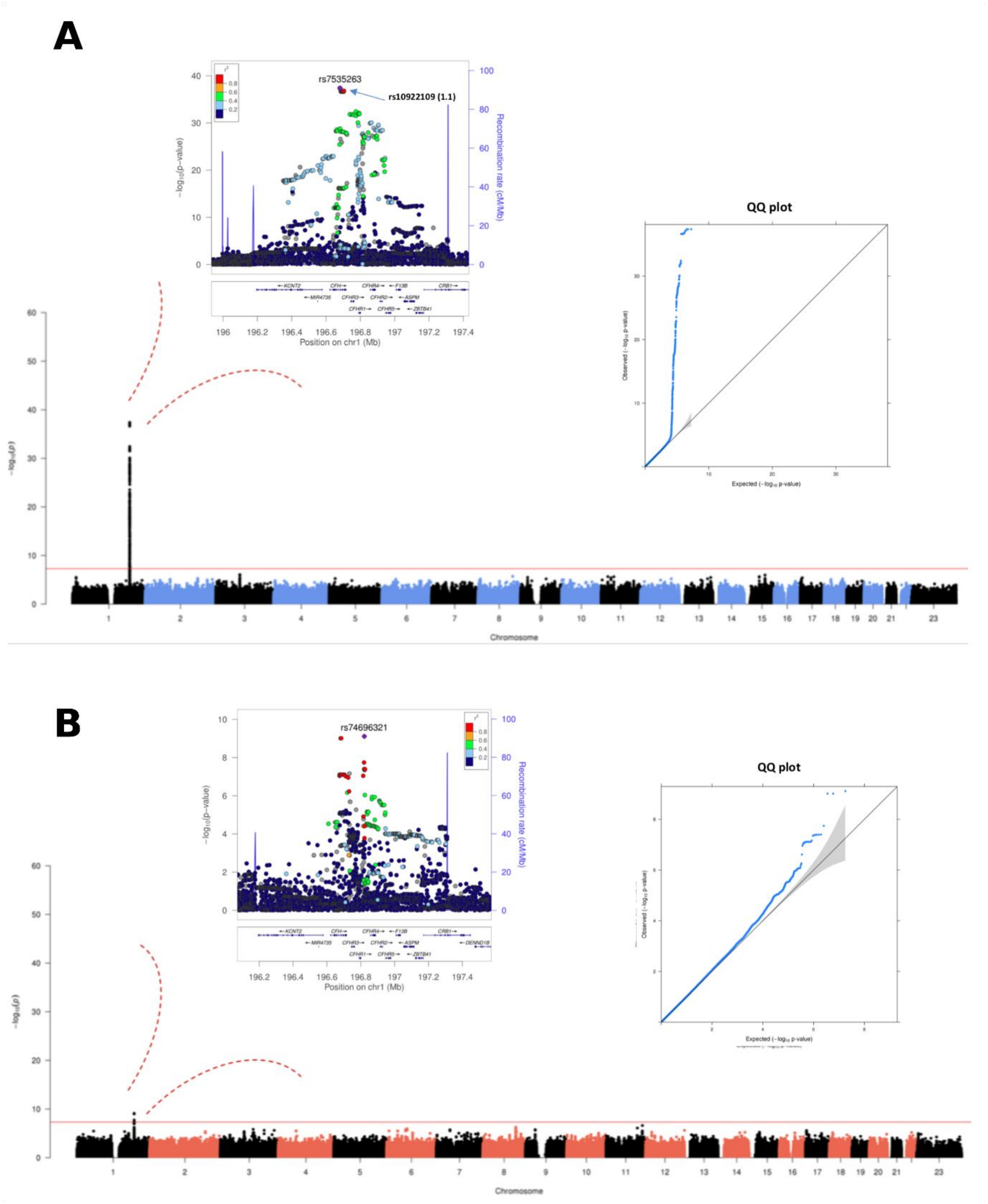

Supplementary Figure 6. GWAS meta-analysis of FHR-4 levels in controls reveals a strong genome-wide significant signal spanning the *CFH* locus.

Each panel shows a Manhattan plot, a regional plot (upper left-hand side) and a quantile-quantile (QQ) plot (upper right-hand side) for the results of the GWAS meta-analysis of FHR-4 levels (Panel A) and FH levels (Panel B). Manhattan plots illustrate P-values for each single variant tested for association with log(levels). Observed -log$_{10}$(P-values) are plotted against the

genomic position of each variant on chromosomes 1–22 plus the X chromosome. The horizontal red line indicates the threshold considered for genome-wide significance (P-value ≤ 5 x 10^{-8}). Regional plots show the only genome-wide association signal observed, i.e., at the *CFH* locus (on chromosome 1q31.3). The most associated variant is denoted by a purple circle and is labelled by its rsID. The other surrounding variants are shown by circles coloured to reflect the extent of LD with the most associated variant (based on 1000 Genomes data, November 2014). A diagram of the genes within the relevant regions is depicted below each plot. Physical positions are based on NCBI RefSeq hg19 human genome reference assembly. QQ plots compare the distribution of the observed test statistics with its expected distribution under the null hypothesis of no association. A marked departure from the null hypothesis (reference line) is seen in the meta-analysis of FHR-4 levels (corresponding to the *CFH* locus). Genomic inflation values (λ) were equal to 1.005 and 0.998 from the GWASs of FHR-4 levels and 0.998 and 0.999 from the GWASs of FH levels, in the Cambridge and EUGENDA studies, respectively.

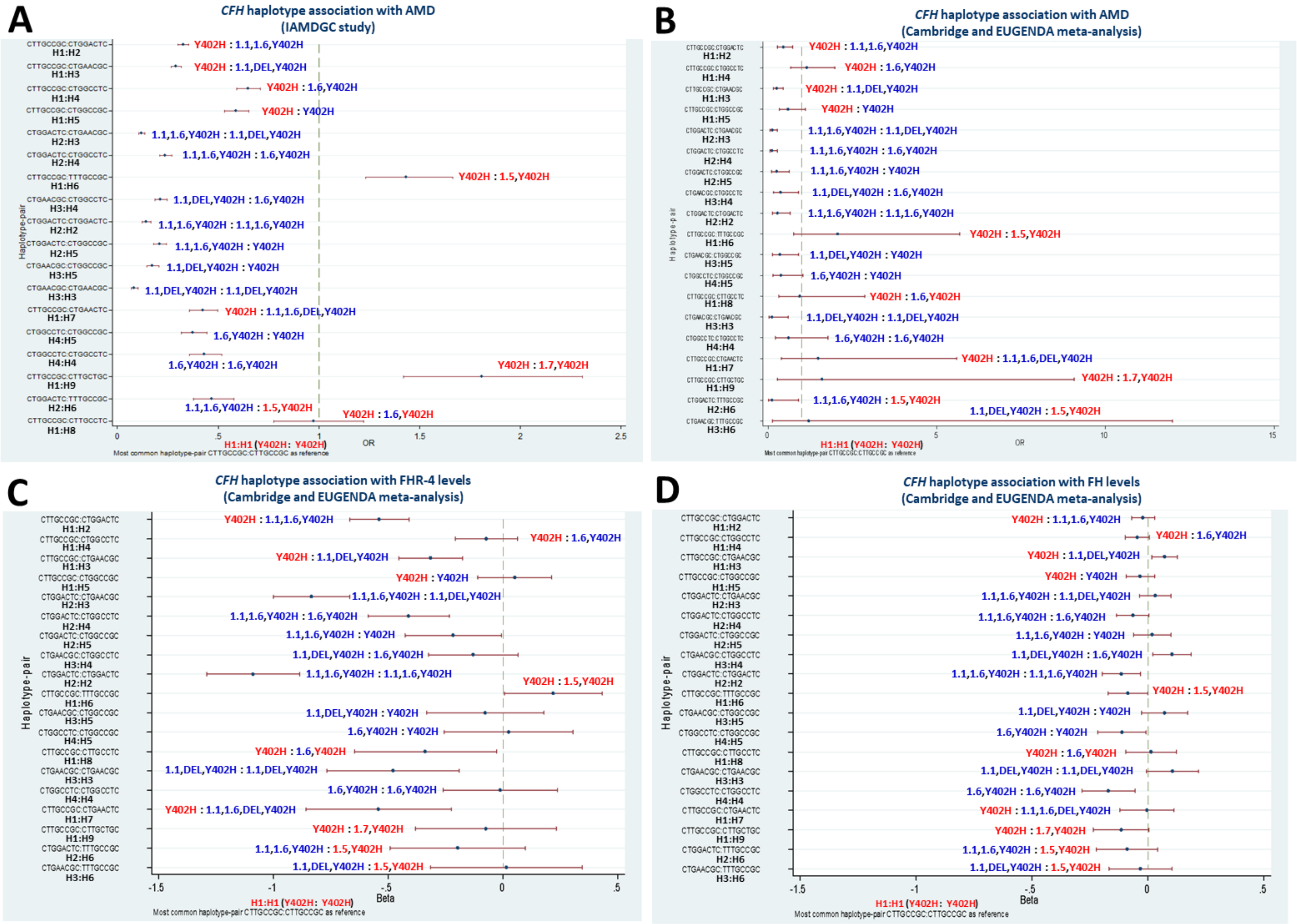

Supplementary Figure 7. Association analyses of the common diplotypes (haplotype pairs, with overall frequency ≥ 1%) formed by the 7

AMD independently associated variants at the *CFH* locus considered in our study and rs6677604 (proxy for the previously reported AMD protective *CFHR1-3* deletion[11]) with AMD, FHR-4 and FH levels.

Panels A and B show the OR (with 95% CI) estimates for the *CFH* diplotype (haplotype-pair) association with AMD in the IAMDGC dataset and the Cambridge and EUGENDA meta-analysis, respectively; panels C and D show the Beta (with 95% CI) estimates for the *CFH* diplotype (haplotype-pair) association with FHR-4 and FH levels, respectively, in the Cambridge and EUGENDA meta-analysis; the haplotype-pair H1:H1 is used as reference. Numerical details together with haplotype-pair frequencies and P-values are given in Supplementary Data 10. The estimates shown in each plot are labelled further according to the presence of the alleles that make each haplotype different from the reference H1, that is indicated with the corresponding IAMDGC association signal numbers (1.1, 1.5-1.7), in red if the allele different from the reference is AMD risk-increasing, in blue if protective; the Y402H label is blue to indicate the presence of the protective allele G of variant 1.2 (rs570618, proxy of Y402H), red for the AMD risk-increasing allele T; finally, the label DEL indicates the presence of the protective allele A of the proxy for the *CFHR1-3* deletion (rs6677604). Source data are provided as a Source Data file.

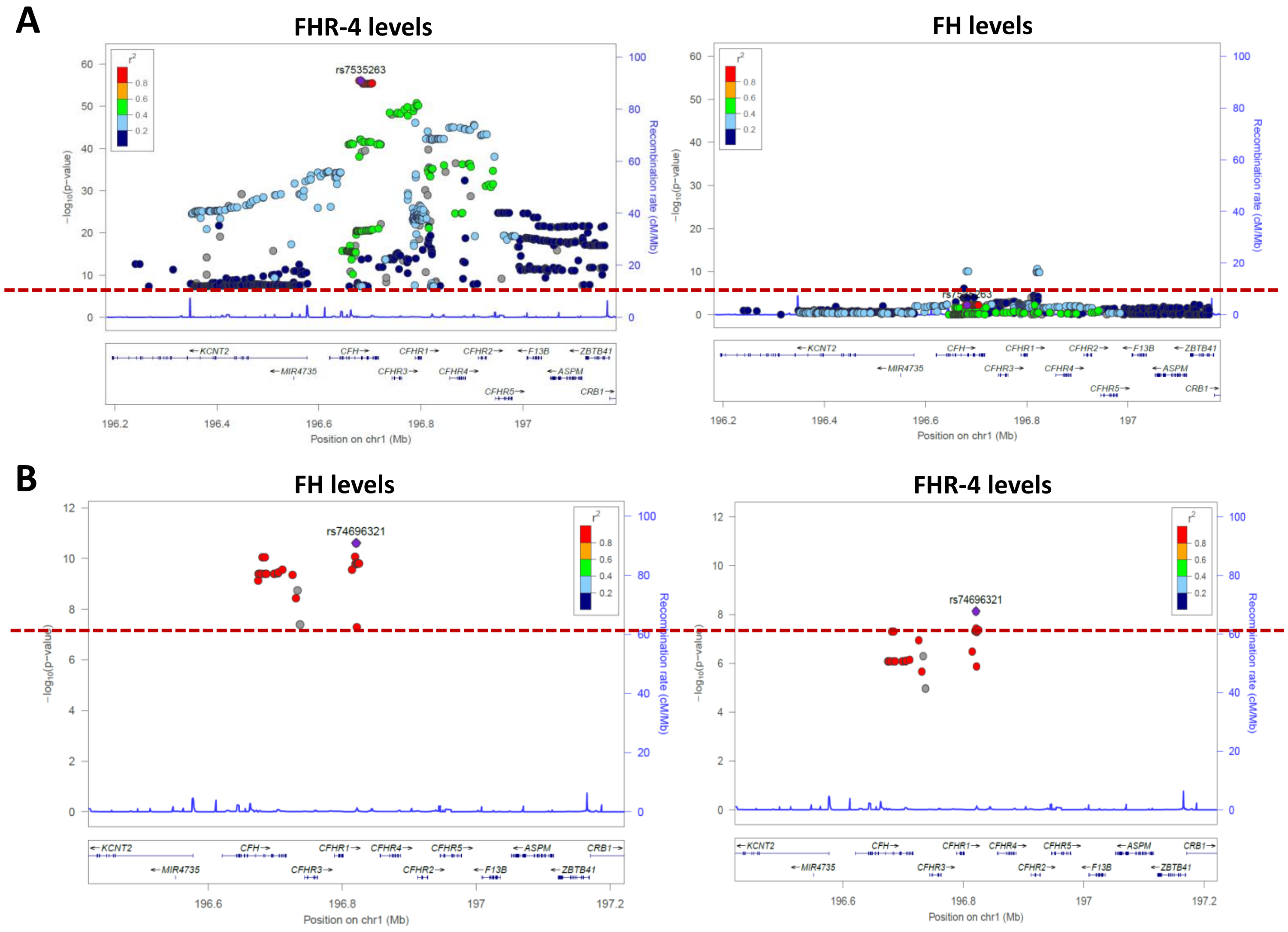

Supplementary Figure 8. FHR-4 and FH levels are dictated by a different genetic architecture.

Regional plots show results from two-cohort (Cambridge and EUGENDA) GWAS meta-analysis of FHR-4 and FH levels only for those variants that showed genome-wide significant (P-value $\leq 5 \times 10^{-8}$) associations with levels of FHR-4 (Panel A) and FH (Panel B). The most associated variant (rs7535263 and rs74696321 for levels of FHR-4 and FH, respectively) is denoted by a purple circle and is labelled by its rsID. The other surrounding variants (811 and 28 for Panel A and B, respectively) are shown by circles coloured to reflect the extent of D with the most associated variant (based on 1000 Genomes data, November 2014). A diagram of the genes within the relevant regions is depicted below each plot. Physical positions are based on NCBI RefSeq hg19 human genome reference assembly.

* ** *** ****

HHHHHHGSSENLYFQGSSGQEVKPCDFPEIQHGGLYYKSLRRLYFPAAAGQSYSYYCDQNF
VTPSGSYWDYIHCTQDGWSPTVPCLRTCSKSDIEIENGFISESSSIYILNKEIQYKCKPGYATAD
GNSSGSITCLQNGWSAQPICIKFCDMPVFENSRAKSNGMRFKLHDTLDYECYDGYEISYGNT
TGSIVCGEDGWSHFPTCYNSSEKCGPPPPISNGDTTSFLLKVYVPQSRVEYQCQSYYELQGSN
YVTCSNGEWSEPPRCIHPCIITEENMNKNNIQLKGKSDIKYYAKTGDTIEFMCKLGYNANTSV
LSFQAVCREGIVEYPRCE

Supplementary Figure 9. Sequence of FHR-4 recombinant protein.

Recombinant FHR-4 gene synthesis was carried out by GenScript using their gene synthesis and protein expression service and is based on the published sequence for the FHR-4B variant of the *CFHR4* gene (UniProt identifier Q92496-3). The original recombinant protein included an N-terminal 6xHis tag (*) followed by, a linker region (**), and a TEV protease cleavage site (***). Removal of the N-terminal His tag results in two non-authentic N-terminal residues (****).

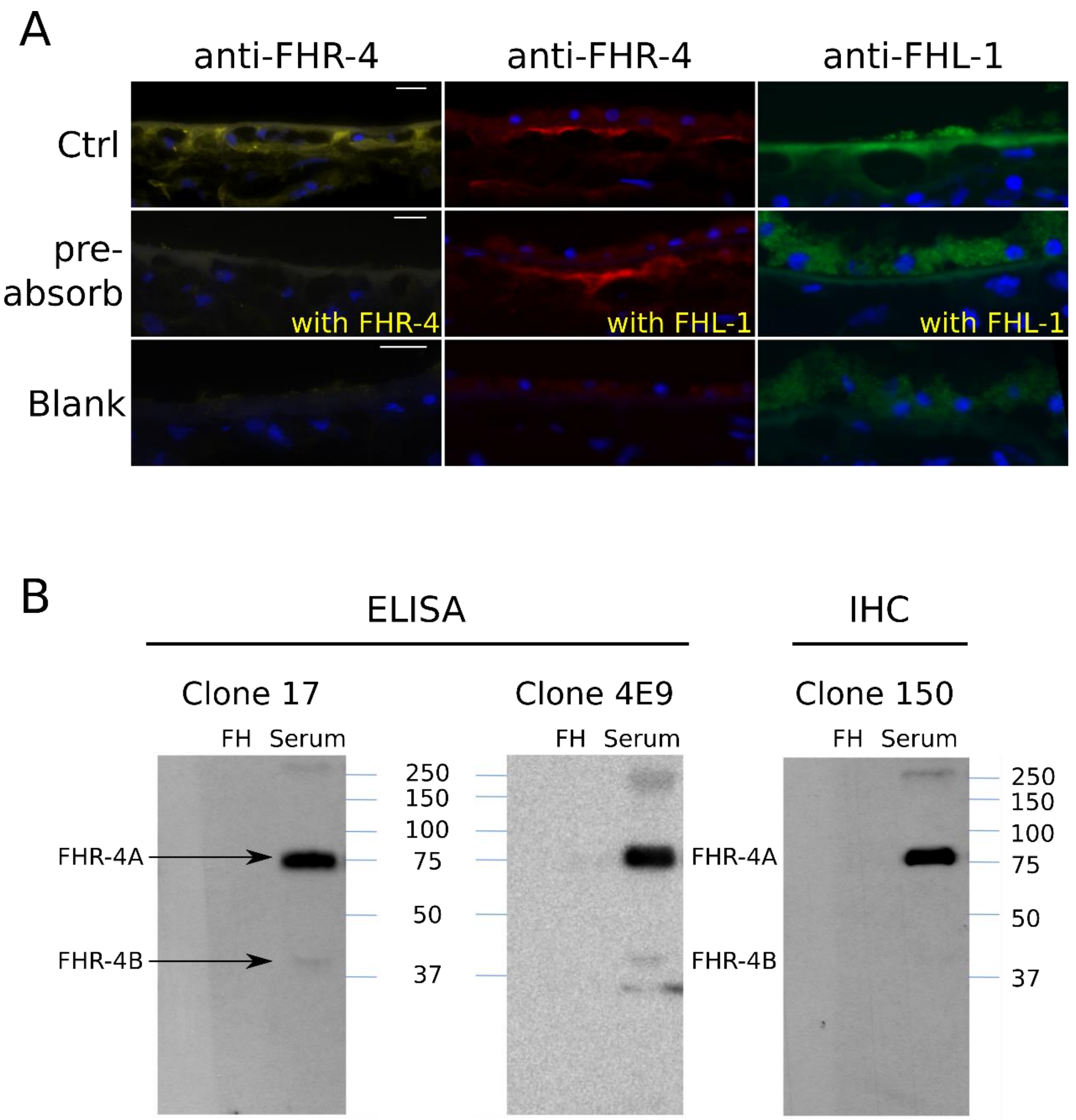

Supplementary Figure 10. Specificity of anti-FHR-4 antibody.

Panel A: the tissue staining specificity of anti-FHR-4 used in our IHC experiments (clone 150) was tested, where the normal 10µg/ml Ab mix used throughout the study was pre-incubated with pure recombinant FHR-4 at a final concentration of 100µg/ml (i.e. 10-fold excess). Staining from the pre-absorption experiments was strikingly similar to the blank controls, where no primary antibody is included. This was repeated with pure FHL-1 protein to demonstrate no cross-reactivity with the antibody existed. The specificity of FHL-1 staining itself with an in-house anti-FHL-1 antibody was also tested, as originally published previously.[12] Panel B: Western blots of non-reduced whole human serum showing three separate clones of anti-FHR-4 antibody with strong reactivity for a band corresponding to FHR-4A, and a faint band corresponding to FHR-4B: the larger FHR-4A has been reported to be the predominant form of FHR-4 in blood.[13] The lanes designated 'FH' had pure factor H protein

loaded to investigate any potential cross-reactivity with the anti-FHR-4 Abs and the full length protein. Source data are provided as a Source Data file.

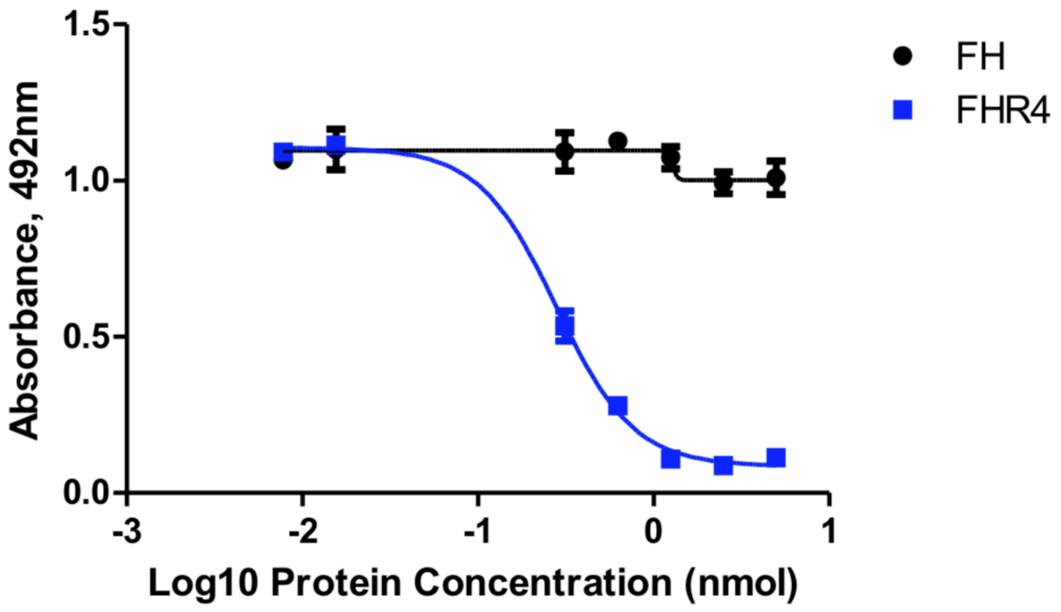

Supplementary Figure 11. Competition ELISA demonstrating specificity of anti-FHR4 antibody clone 150 for FHR-4 over FH.

Immobilised FHR-4 protein was detected by the addition of a saturating dose of the anti-FHR-4 monoclonal antibody used in IHC experiments and ELISA (clone 150). Serial dilutions of either FH (black line) or FHR-4 (blue line) were added in solution together with the anti-FHR-4 antibody. Bound anti-FHR-4 was detected by the addition of anti-mouse IgG HRP-conjugated secondary antibody. Bound secondary antibody detected by addition of OPD substrate and measurement of absorbance at OD492nm. For each data point n=3 and error bars shown are standard error of the mean of the triplicates. Source data are provided as a Source Data file.

Supplementary Note 1

List of the International Age-related Macular Degeneration Genomics Consortium (IAMDGC) members

The list reflects the author list of the previous IAMDGC publication by Fritsche *et al.*, 2016.[9]

Lars G Fritsche[1], Wilmar Igl[2], Jessica N Cooke Bailey[3], Felix Grassmann[4], Sebanti Sengupta[1], Jennifer L Bragg-Gresham[1,5], Kathryn P Burdon[6], Scott J Hebbring[7], Cindy Wen[8], Mathias Gorski[2], Ivana K Kim[9], David Cho[10], Donald Zack[11-15], Eric Souied[16], Hendrik P N Scholl[11,17], Elisa Bala[18], Kristine E Lee[19], David J Hunter[20,21], Rebecca J Sardell[22], Paul Mitchell[23], Joanna E Merriam[24], Valentina Cipriani[25,26], Joshua D Hoffman[27], Tina Schick[28], Yara T E Lechanteur[29], Robyn H Guymer[30], Matthew P Johnson[31], Yingda Jiang[32], Chloe M Stanton[33], Gabriëlle H S Buitendijk[34,35], Xiaowei Zhan[1,36,37], Alan M Kwong[1], Alexis Boleda[38], Matthew Brooks[38], Linn Gieser[38], Rinki Ratnapriya[38], Kari E Branham[39], Johanna R Foerster[1], John R Heckenlively[39], Mohammad I Othman[39], Brendan J Vote[6], Helena Hai Liang[30], Emmanuelle Souzeau[40], Ian L McAllister[41], Timothy Isaacs[41], Janette Hall[40], Stewart Lake[40], David A Mackey[6,30,41], Ian J Constable[41], Jamie E Craig[40], Terrie E Kitchner[7], Zhenglin Yang[42,43], Zhiguang Su[44], Hongrong Luo[8], Daniel Chen[8], Hong Ouyang[8], Ken Flagg[8], Danni Lin[8], Guanping Mao[8], Henry Ferreyra[8], Klaus Stark[2], Claudia N von Strachwitz[45], Armin Wolf[46], Caroline Brandl[2,4,47], Guenther Rudolph[46], Matthias Olden[2], Margaux A Morrison[48], Denise J Morgan[48], Matthew Schu[49-53], Jeeyun Ahn[54], Giuliana Silvestri[55], Evangelia E Tsironi[56], Kyu Hyung Park[57], Lindsay A Farrer[49-53], Anton Orlin[58], Alexander Brucker[59], Mingyao Li[60], Christine A Curcio[61], Saddek Mohand-Saïd[62-65], José-Alain Sahel[25,62-67], Isabelle Audo[62-64,68], Mustapha Benchaboune[65], Angela J Cree[69], Christina A Rennie[70], Srinivas V Goverdhan[69], Michelle Grunin[71], Shira Hagbi-Levi[71], Peter Campochiaro[11,13], Nicholas Katsanis[72-74], Frank G Holz[17], Frédéric Blond[62-64], Hélène Blanché[75], Jean-François Deleuze[75,76], Robert P Igo Jr[3], Barbara Truitt[3], Neal S Peachey[18,77], Stacy M Meuer[19], Chelsea E Myers[19], Emily L Moore[19], Ronald Klein[19], Michael A Hauser[78-80], Eric A Postel[78], Monique D Courtenay[22], Stephen G Schwartz[81], Jaclyn L Kovach[81], William K Scott[22], Gerald Liew[23], Ava G Tan[23], Bamini Gopinath[23], John C Merriam[24], R Theodore Smith[24,82], Jane C Khan[41,83,84], Humma Shahid[84,85], Anthony T Moore[25,26,86], J Allie McGrath[27], Reneé Laux[3], Milam A Brantley Jr[87], Anita Agarwal[87], Lebriz Ersoy[28], Albert Caramoy[28], Thomas Langmann[28], Nicole T M Saksens[29], Eiko K de Jong[29], Carel B Hoyng[29], Melinda S Cain[30], Andrea J Richardson[30], Tammy M

Martin[88], John Blangero[31], Daniel E Weeks[32,89], Bal Dhillon[90], Cornelia M van Duijn[35], Kimberly F Doheny[91], Jane Romm[91], Caroline C W Klaver[34,35], Caroline Hayward[33], Michael B Gorin[92,93], Michael L Klein[88], Paul N Baird[30], Anneke I den Hollander[29,94], Sascha Fauser[28], John R W Yates[25,26,84], Rando Allikmets[24,95], Jie Jin Wang[23], Debra A Schaumberg[20,96,97], Barbara E K Klein[19], Stephanie A Hagstrom[77], Itay Chowers[71], Andrew J Lotery[69], Thierry Léveillard[62-64], Kang Zhang[8,44], Murray H Brilliant[7], Alex W Hewitt[6,30,41], Anand Swaroop[38], Emily Y Chew[98], Margaret A Pericak-Vance[22], Margaret DeAngelis[48], Dwight Stambolian[10], Jonathan L Haines[3,99], Sudha K Iyengar[3], Bernhard H F Weber[4], Gonçalo R Abecasis[1] & Iris M Heid[2]

IAMDGC members' affiliations

[1]Center for Statistical Genetics, Department of Biostatistics, University of Michigan, Ann Arbor, Michigan, USA. [2]Department of Genetic Epidemiology, University of Regensburg, Regensburg, Germany. [3]Department of Epidemiology and Biostatistics, Case Western Reserve University School of Medicine, Cleveland, Ohio, USA. [4]Institute of Human Genetics, University of Regensburg, Regensburg, Germany. [5]Kidney Epidemiology and Cost Center, Department of Internal Medicine–Nephrology, University of Michigan, Ann Arbor, Michigan, USA. [6]School of Medicine, Menzies Research Institute Tasmania, University of Tasmania, Hobart, Tasmania, Australia. [7]Center for Human Genetics, Marshfield Clinic Research Foundation, Marshfield, Wisconsin, USA. [8]Department of Ophthalmology, University of California, San Diego and Veterans Affairs San Diego Health System, La Jolla, California, USA. [9]Retina Service, Massachusetts Eye and Ear, Department of Ophthalmology, Harvard Medical School, Boston, Massachusetts, USA. [10]Department of Ophthalmology, Perelman School of Medicine, University of Pennsylvania, Philadelphia, Pennsylvania, USA. [11]Department of Ophthalmology, Wilmer Eye Institute, Johns Hopkins University School of Medicine, Baltimore, Maryland, USA. [12]Department of Molecular Biology and Genetics, Johns Hopkins University School of Medicine, Baltimore, Maryland, USA. [13]Department of Neuroscience, Johns Hopkins University School of Medicine, Baltimore, Maryland, USA. [14]Institute of Genetic Medicine, Johns Hopkins University School of Medicine, Baltimore, Maryland, USA. [15]Institue de la Vision, Université Pierre et Marie Curie, Paris, France. [16]Hôpital Intercommunal de Créteil, Hôpital Henri Mondor, Université Paris Est Créteil, Créteil, France. [17]Department of Ophthalmology, University of Bonn, Bonn, Germany. [18]Louis Stokes Cleveland Veterans Affairs Medical Center, Cleveland, Ohio, USA. [19]Department of

Ophthalmology and Visual Sciences, University of Wisconsin, Madison, Wisconsin, USA. [20]Department of Epidemiology, Harvard School of Public Health, Boston, Massachusetts, USA. [21]Department of Nutrition, Harvard School of Public Health, Boston, Massachusetts, USA. [22]John P. Hussman Institute for Human Genomics, Miller School of Medicine, University of Miami, Miami, Florida, USA. [23]Centre for Vision Research, Department of Ophthalmology and Westmead Millennium Institute for Medical Research, University of Sydney, Sydney, New South Wales, Australia. [24]Department of Ophthalmology, Columbia University, New York, New York, USA. [25]University College London Institute of Ophthalmology, University College London, London, UK. [26]Moorfields Eye Hospital, London, UK. [27]Center for Human Genetics Research, Vanderbilt University Medical Center, Nashville, Tennessee, USA. [28]Department of Ophthalmology, University Hospital of Cologne, Cologne, Germany. [29]Department of Ophthalmology, Radboud University Medical Centre, Nijmegen, the Netherlands. [30]Centre for Eye Research Australia, University of Melbourne, Royal Victorian Eye and Ear Hospital, East Melbourne, Victoria, Australia. [31]South Texas Diabetes and Obesity Institute, School of Medicine, University of Texas Rio Grande Valley, Brownsville, Texas, USA. [32]Department of Biostatistics, Graduate School of Public Health, University of Pittsburgh, Pittsburgh, Pennsylvania, USA. [33]Medical Research Council (MRC) Human Genetics Unit, Institute of Genetics and Molecular Medicine, University of Edinburgh, Edinburgh, UK. [34]Department of Ophthalmology, Erasmus Medical Center, Rotterdam, the Netherlands. [35]Department of Epidemiology, Erasmus Medical Center, Rotterdam, the Netherlands. [36]Quantitative Biomedical Research Center, Department of Clinical Science, University of Texas Southwestern Medical Center, Dallas, Texas, USA. [37]Center for the Genetics of Host Defense, University of Texas Southwestern Medical Center, Dallas, Texas, USA. [38]Neurobiology, Neurodegeneration and Repair Laboratory (N-NRL), National Eye Institute, US National Institutes of Health, Bethesda, Maryland, USA. [39]Department of Ophthalmology and Visual Sciences, University of Michigan, Kellogg Eye Center, Ann Arbor, Michigan, USA. [40]Department of Ophthalmology, Flinders Medical Centre, Flinders University, Adelaide, South Australia, Australia. [41]Centre for Ophthalmology and Visual Science, Lions Eye Institute, University of Western Australia, Perth, Western Australia, Australia. [42]Sichuan Provincial Key Laboratory for Human Disease Gene Study, Hospital of the University of Electronic Science and Technology of China and Sichuan Provincial People's Hospital, Chengdu, China. [43]Sichuan Translational Medicine Hospital, Chinese Academy of Sciences, Chengdu, China. [44]Molecular Medicine Research Center, State Key Laboratory of Biotherapy, West China Hospital, Sichuan University, Chengdu, China. [45]EyeCentre

Southwest, Stuttgart, Germany. [46]University Eye Clinic, Ludwig Maximilians University, Munich, Germany. [47]Department of Ophthalmology, University Hospital Regensburg, Regensburg, Germany. [48]Department of Ophthalmology and Visual Sciences, University of Utah, Salt Lake City, Utah, USA. [49]Department of Medicine (Biomedical Genetics), Boston University Schools of Medicine and Public Health, Boston, Massachusetts, USA. [50]Department of Ophthalmology, Boston University Schools of Medicine and Public Health, Boston, Massachusetts, USA. [51]Department of Neurology, Boston University Schools of Medicine and Public Health, Boston, Massachusetts, USA. [52]Department of Epidemiology, Boston University Schools of Medicine and Public Health, Boston, Massachusetts, USA. [53]Department of Biostatistics, Boston University Schools of Medicine and Public Health, Boston, Massachusetts, USA. [54]Department of Ophthalmology, Seoul Metropolitan Government Seoul National University Boramae Medical Center, Seoul, Republic of Korea. [55]Centre for Experimental Medicine, Queen's University, Belfast, UK. [56]Department of Ophthalmology, University of Thessaly, School of Medicine, Larissa, Greece. [57]Department of Ophthalmology, Seoul National University Bundang Hospital, Seongnam, Republic of Korea. [58]Department of Ophthalmology, Weill Cornell Medical College, New York, New York, USA. [59]Scheie Eye Institute, Department of Ophthalmology, University of Pennsylvania Perelman School of Medicine, Philadelphia, Pennsylvania, USA. [60]Department of Biostatistics and Epidemiology, University of Pennsylvania Perelman School of Medicine, Philadelphia, Pennsylvania, USA. [61]Department of Ophthalmology, University of Alabama at Birmingham, Birmingham, Alabama, USA. [62]INSERM, Paris, France. [63]Institut de la Vision, Department of Genetics, Paris, France. [64]Centre National de la Recherche Scientifique (CNRS), Paris, France. [65]Centre Hospitalier National d'Ophtalmologie des Quinze-Vingts, Paris, France. [66]Fondation Ophtalmologique Adolphe de Rothschild, Paris, France. [67]Académie des Sciences–Institut de France, Paris, France. [68]Department of Molecular Genetics, Institute of Ophthalmology, London, UK. [69]Clinical and Experimental Sciences, Faculty of Medicine, University of Southampton, Southampton, UK. [70]University Hospital Southampton, Southampton, UK. [71]Department of Ophthalmology, Hadassah Hebrew University Medical Center, Jerusalem, Israel. [72]Center for Human Disease Modeling, Duke University, Durham, North Carolina, USA. [73]Department of Cell Biology, Duke University, Durham, North Carolina, USA. [74]Department of Pediatrics, Duke University, Durham, North Carolina, USA. [75]Centre d'Etude du Polymorphisme Humain (CEPH) Fondation Jean Dausset, Paris, France. [76]Commissariat à l'Energie Atomique et aux Energies Alternatives (CEA), Institut de Génomique, Centre National de Génotypage, Evry, France. [77]Cole Eye Institute, Cleveland Clinic, Cleveland,

Ohio, USA. [78]Department of Ophthalmology, Duke University Medical Center, Durham, North Carolina, USA. [79]Department of Medicine, Duke University Medical Center, Durham, North Carolina, USA. [80]Duke Molecular Physiology Institute, Duke University Medical Center, Durham, North Carolina, USA. [81]Bascom Palmer Eye Institute, University of Miami Miller School of Medicine, Naples, Florida, USA. [82]Department of Ophthalmology, New York University School of Medicine, New York, New York, USA. [83]Department of Ophthalmology, Royal Perth Hospital, Perth, Western Australia, Australia. [84]Department of Medical Genetics, Cambridge Institute for Medical Research, University of Cambridge, Cambridge, UK. [85]Department of Ophthalmology, Cambridge University Hospitals National Health Service (NHS) Foundation Trust, Cambridge, UK. [86]Department of Ophthalmology, University of California San Francisco Medical School, San Francisco, California, USA. [87]Department of Ophthalmology and Visual Sciences, Vanderbilt University, Nashville, Tennessee, USA. [88]Casey Eye Institute, Oregon Health and Science University, Portland, Oregon, USA. [89]Department of Human Genetics, Graduate School of Public Health, University of Pittsburgh, Pittsburgh, Pennsylvania, USA. [90]School of Clinical Sciences, University of Edinburgh, Edinburgh, UK. [91]Center for Inherited Disease Research (CIDR) Institute of Genetic Medicine, Johns Hopkins University School of Medicine, Baltimore, Maryland, USA. [92]Department of Ophthalmology, David Geffen School of Medicine, Stein Eye Institute, University of California, Los Angeles, Los Angeles, California, USA. [93]Department of Human Genetics, David Geffen School of Medicine, University of California, Los Angeles, Los Angeles, California, USA. [94]Department of Human Genetics, Radboud University Medical Centre, Nijmegen, the Netherlands. [95]Department of Pathology and Cell Biology, Columbia University, New York, New York, USA. [96]Center for Translational Medicine, Moran Eye Center, University of Utah School of Medicine, Salt Lake City, Utah, USA. [97]Division of Preventive Medicine, Brigham and Women’s Hospital, Harvard Medical School, Boston, Massachusetts, USA. [98]Division of Epidemiology and Clinical Applications, Clinical Trials Branch, National Eye Institute, US National Institutes of Health, Bethesda, Maryland, USA. [99]Institute for Computational Biology, Case Western Reserve University School of Medicine, Cleveland, Ohio, USA.

Supplementary References

1. Bird, A.C., *et al.* An international classification and grading system for age-related maculopathy and age-related macular degeneration. *Surv Ophthalmol* **39**, 367-374 (1995).
2. Mokwa, N.F., *et al.* Grading of Age-Related Macular Degeneration: Comparison between Color Fundus Photography, Fluorescein Angiography, and Spectral Domain Optical Coherence Tomography. *J Ophthalmol* **2013**(2013).
3. Wagner, A.H., *et al.* Exon-level expression profiling of ocular tissues. *Exp Eye Res* **111**, 105-111 (2013).
4. Kim, E.J., *et al.* Complete Transcriptome Profiling of Normal and Age-Related Macular Degeneration Eye Tissues Reveals Dysregulation of Anti-Sense Transcription. *Sci Rep* **8**, 3040 (2018).
5. Li, M.Y., *et al.* Comprehensive analysis of gene expression in human retina and supporting tissues. *Hum Mol Genet* **23**, 4001-4014 (2014).
6. Whitmore, S.S., *et al.* Altered gene expression in dry age-related macular degeneration suggests early loss of choroidal endothelial cells. *Mol Vis* **19**, 2274-2297 (2013).
7. Strunnikova, N.V., *et al.* Transcriptome analysis and molecular signature of human retinal pigment epithelium. *Hum Mol Genet* **19**, 2468-2486 (2010).
8. GTEx Consortium. Human genomics. The Genotype-Tissue Expression (GTEx) pilot analysis: multitissue gene regulation in humans. *Science* **348**, 648-660 (2015).
9. Fritsche, L.G., *et al.* A large genome-wide association study of age-related macular degeneration highlights contributions of rare and common variants. *Nat Genet* **48**, 134-143 (2016).
10. Raychaudhuri, S., *et al.* A rare penetrant mutation in CFH confers high risk of age-related macular degeneration. *Nat Genet* **43**, 1232-1236 (2011).
11. Hughes, A.E., *et al.* A common CFH haplotype, with deletion of CFHR1 and CFHR3, is associated with lower risk of age-related macular degeneration. *Nat Genet* **38**, 1173-1177 (2006).
12. Clark, S.J., *et al.* Identification of Factor H-like Protein 1 as the Predominant Complement Regulator in Bruch's Membrane: Implications for Age-Related Macular Degeneration. *J Immunol* **193**, 4962-4970 (2014).
13. Pouw, R.B., *et al.* Complement Factor H-Related Protein 4A Is the Dominant Circulating Splice Variant of CFHR4. *Front Immunol* **9**(2018).
