# Supplementary material for: Increased circulating levels of Factor H-Related Protein 4 are strongly associated with age-related macular degeneration
Source: Nat Commun. 2020 Feb 7;11:778. doi: 10.1038/s41467-020-14499-3 (PMC7005798; doi:10.1038/s41467-020-14499-3)
Supplement: Supplementary file 3 — Description of Additional Supplementary Information [file 41467_2020_14499_MOESM3_ESM.pdf]

Description of Additional Supplementary Files

File Name: Supplementary Data 1
Description: Single-variant association analyses with AMD for the 8 AMD independently associated variants at the CFH locus from the IAMDGC study (Fritsche et al. 2016).

File Name: Supplementary Data 2
Description: Single-variant association analyses with FHR-4/FH levels for the 8 AMD independently associated variants at the CFH locus from the IAMDGC study (Fritsche et al. 2016).

File Name: Supplementary Data 3
Description: Association results for the variants showing a P-value ≤ 10-6 in the GWAS meta-analysis of FHR-4 levels using controls only.

File Name: Supplementary Data 4
Description: Association results for the variants showing a P-value ≤ 10-6 in the GWAS meta-analysis of FHR-4 levels using all samples, further adjusting for AMD status.

File Name: Supplementary Data 5
Description: Linkage disequilibrium measures (R2 and D') for the variants showing the top association signals in the GWAS meta-analysis of FHR-4 levels using all samples.

File Name: Supplementary Data 6
Description: Association results for the variants showing a P-value ≤ 10-6 in the GWAS meta-analysis of FH levels using controls only.

File Name: Supplementary Data 7
Description: Association results for the variants showing a P-value ≤ 10-6 in the GWAS meta-analysis of FH levels using all samples, further adjusting for AMD status.

File Name: Supplementary Data 8
Description: Linkage disequilibrium measures (R2 and D') for the variants showing the genome-wide association signals in the GWAS meta-analysis of FH levels using all samples.

File Name: Supplementary Data 9
Description: Association analyses of the common haplotypes (with overall frequency ≥ 1%) formed by the 7 AMD independently associated variants at the CFH locus considered in our study and rs6677604 (proxy for the previously reported AMD protective CFHR1-3 deletion (Hughes et al. 2006)) with AMD, FHR-4 and FH levels.

File Name: Supplementary Data 10
Description: Association analyses of the common diplotypes (haplotype pairs, with overall frequency ≥ 1%) formed by the 7 AMD independently associated variants at the CFH locus considered in our study and rs6677604 (proxy for the previously reported AMD protective CFHR1-3 deletion (Hughes et al. 2006)) with AMD, FHR-4 and FH levels.

File Name: Supplementary Data 11

Description: Association analyses of the haplotypes formed by rs10922109 (1.1) and rs61818925 (1.6) with AMD, FHR-4 and FH levels.
