# Supplementary material for: Increased circulating levels of Factor H-Related Protein 4 are strongly associated with age-related macular degeneration
Source: Nat Commun. 2020 Feb 7;11:778. doi: 10.1038/s41467-020-14499-3 (PMC7005798; doi:10.1038/s41467-020-14499-3)
Supplement: Supplementary file 15 — Reporting Summary [file 41467_2020_14499_MOESM15_ESM.pdf]

nature research

Corresponding author(s): Valentina Cipriani (v.cipriani@qmul.ac.uk) Simon Clark (simon.clark-3@manchester.ac.uk)

Last updated by author(s): Aug 1, 2019

Reporting Summary

Nature Research wishes to improve the reproducibility of the work that we publish. This form provides structure for consistency and transparency in reporting. For further information on Nature Research policies, see Authors & Referees and the Editorial Policy Checklist .

Statistics

For all statistical analyses, confirm that the following items are present in the figure legend, table legend, main text, or Methods section.

n/a	Confirmed	
☐	☒	The exact sample size (*n*) for each experimental group/condition, given as a discrete number and unit of measurement
☐	☒	A statement on whether measurements were taken from distinct samples or whether the same sample was measured repeatedly
☐	☒	The statistical test(s) used AND whether they are one- or two-sided<br>*Only common tests should be described solely by name; describe more complex techniques in the Methods section.*
☐	☒	A description of all covariates tested
☐	☒	A description of any assumptions or corrections, such as tests of normality and adjustment for multiple comparisons
☐	☒	A full description of the statistical parameters including central tendency (e.g. means) or other basic estimates (e.g. regression coefficient) AND variation (e.g. standard deviation) or associated estimates of uncertainty (e.g. confidence intervals)
☐	☒	For null hypothesis testing, the test statistic (e.g. *F*, *t*, *r*) with confidence intervals, effect sizes, degrees of freedom and *P* value noted<br>*Give P values as exact values whenever suitable.*
☒	☐	For Bayesian analysis, information on the choice of priors and Markov chain Monte Carlo settings
☒	☐	For hierarchical and complex designs, identification of the appropriate level for tests and full reporting of outcomes
☐	☒	Estimates of effect sizes (e.g. Cohen's *d*, Pearson's *r*), indicating how they were calculated

Our web collection on statistics for biologists contains articles on many of the points above.

Software and code

Policy information about availability of computer code

Data collection	No software was used for data collection, only analysis (see below)
Data analysis	Single-variant analyses and haplotype-based association analyses were conducted using Stata software, version 13.1 (StataCorp), with tobit and regress commands; and ipdmetan and mvmeta commands were used for conducting meta-analyses of individual participant data. The GWASs were carried out using EPACTS software (http://genome.sph.umich.edu/wiki/EPACTS) and Wald tests were performed on the variant genotypes coded as 0, 1 and 2 according to the number of minor alleles for the directly typed variants or allele dosages for the imputed variants. Effect size estimates and standard errors of single variants seen in both cohorts were subsequently combined in a fixed-effect meta-analysis using METAL software (https://genome.sph.umich.edu/wiki/METAL_Documentation). Manhattan and Q-Q plots were generated using the qqman R package (version 0.1.2). Regional plots of association were generated using LocusZoom (version v0.4.8). Linkage disequilibrium measures (R2 and D') were calculated using LDlink (https://ldlink.nci.nih.gov/), based on the European (EUR) population genotype data originates from Phase 3 (Version 5) of the 1000 Genomes Project.

For manuscripts utilizing custom algorithms or software that are central to the research but not yet described in published literature, software must be made available to editors/reviewers. We strongly encourage code deposition in a community repository (e.g. GitHub). See the Nature Research guidelines for submitting code & software for further information.

Data

Policy information about availability of data

All manuscripts must include a data availability statement. This statement should provide the following information, where applicable:

- Accession codes, unique identifiers, or web links for publicly available datasets
- A list of figures that have associated raw data
- A description of any restrictions on data availability

The summary statistics for the GWAS meta-analyses of FHR-4 and FH levels will be available through the GWAS Catalog, https://www.ebi.ac.uk/gwas/, [accession codes will be available before publication].

The Gene Expression Omnibus datasets used for the gene expression analyses are available at: https://www.ncbi.nlm.nih.gov/geo/query/acc.cgi?acc=GSE18811, dataset name: GSE18811; https://www.ncbi.nlm.nih.gov/geo/query/acc.cgi?acc=GSE41102, dataset name: GSE41102; https://www.ncbi.nlm.nih.gov/geo/query/acc.cgi?acc=GSE50195, dataset name: GSE50195; https://www.ncbi.nlm.nih.gov/geo/query/acc.cgi?acc=GSE94437, dataset name: GSE94437; https://www.ncbi.nlm.nih.gov/geo/query/acc.cgi?acc=GSE99248, dataset name: GSE:99248.
The Genotype-Tissue Expression (GTEx) Project datasets used for the gene expression analyses were obtained from the GTEx Portal, https://gtexportal.org/home/multiGeneQueryPage (4/4/2018), dataset dbGaP accession number phs000424.v8.p2; the GTEx Project was supported by the Common Fund of the Office of the Director of the National Institutes of Health, and by NCI, NHGRI, NHLBI, NIDA, NIMH, and NINDS.
The source data underlying Figs. 1, 2B-I, 3B, 4, 5A, 6A-B and Supplementary Figs. 2A, 5, 6, 7A-D, 8, 10A, 11 are provided as a Source Data file.
All other datasets generated in the current study are available from the corresponding authors upon reasonable request.

Field-specific reporting

Please select the one below that is the best fit for your research. If you are not sure, read the appropriate sections before making your selection.

[x] Life sciences [ ] Behavioural & social sciences [ ] Ecological, evolutionary & environmental sciences

For a reference copy of the document with all sections, see nature.com/documents/nr-reporting-summary-flat.pdf

Life sciences study design

All studies must disclose on these points even when the disclosure is negative.

Sample size

Biochemical analysis: SPR; C3b breakdown assays; C3b breakdown inhibition assays - all data comprised of individual triplicate runs.
Immunohistochemistry: frozen human eye tissue sections from ten AMD donors and ten non-AMD donors were used for the localisation studies of FHR-4, C3b and Collagen-IV.

Data exclusions

No data excluded from study

Replication

All data described here was reproduced successfully more than twice

Randomization

Biochemical analysis: SPR; C3b breakdown assays; C3b breakdown inhibition assays - no randomisation because these are direct measurement assays using purified components.
Immunohistochemistry: Randomisation was not possible because the lab member was also responsible for accessing the desired AMD vs healthy tissue from the eye tissue repository

Blinding

Biochemical analysis: SPR; C3b breakdown assays; C3b breakdown inhibition assays - no blinding was necessary.
Immunohistochemistry: Staining intensities and patterns of human tissue sections were analyzed blind by an independent team member and not the individual who performed the experiment.

Reporting for specific materials, systems and methods

We require information from authors about some types of materials, experimental systems and methods used in many studies. Here, indicate whether each material, system or method listed is relevant to your study. If you are not sure if a list item applies to your research, read the appropriate section before selecting a response.

Materials & experimental systems

n/a	Involved in the study	
[ ]	[x]	Antibodies
[x]	[ ]	Eukaryotic cell lines
[x]	[ ]	Palaeontology
[x]	[ ]	Animals and other organisms
[ ]	[x]	Human research participants
[x]	[ ]	Clinical data

Methods

n/a	Involved in the study	
[x]	[ ]	ChIP-seq
[x]	[ ]	Flow cytometry
[x]	[ ]	MRI-based neuroimaging

Antibodies

Antibodies used

Monoclonal anti-FHR-4 (clones 4E9 and 17 (sandwich ELISA) and 150 (IHC)) were made in house: full description of methodologies is provided in the manuscript.
Polyclonal anti-FHL-1 was made using the Mimotopes antibody generation service as described in Clark et al. (2014) J. Immunol. 193, 4962.
Polyclonal anti-C3/C3b was purchased commercially from Proteintech Group, USA (cat. no. 21337-1-AP).
Polyclonal anti-Collagen IV was purchased commercially from 2B Scientific Ltd., Oxford, UK (Cat. no. 600-401-106S)

Validation	Monoclonal anti-FHR-4 antibody clones 4E9, 17 and 150 were all tested in western blot against purified recombinant FHR-4 and whole human serum, using FH as a negative control (see Supplementary Fig. 10). Clone 150, subsequently used in IHC experiments, was further validated by pre-absorption with purified FHR-4 to quench fluorescent binding signal. This was also performed with FHL-1 to demonstrate no cross-reactivity with endogenous FHL-1 in the samples. Additional ELISA have been performed to show that excessive FH levels do not interfere with the recognition of FHR-4 by clone 150 (used in IHC experiments).

Human research participants

Policy information about studies involving human research participants

Population characteristics	Two advanced AMD case-control cohorts:<br>Cambridge: 214 controls, 304 cases; EUGENDA: 308 controls, 180 cases;<br>corresponding Mean age, ys (SD): 75.2 (8.0), 74.1 (8.3); 70.0 (6.5), 79.3 (8.6);<br>corresponding Male %: 36.5, 47.0; 42.9, 42.2.<br>All subjects of European ancestry.
Recruitment	The Cambridge AMD study is a case-control study with subjects recruited from the southeast and northwest of England between 2002-2006. All affected subjects had choroidal neovascularization (CNV) and/or geographic atrophy (GA). Controls were spouses, partners or friends of index patients (details on exclusion criteria and grading are in the Methods section of Supplementary Information). Blood samples were obtained at the time of interview; EDTA and lithium-heparin plasma samples were used for DNA extraction and FHR-4/FH measurements respectively. The European Genetic Database (EUGENDA) created for clinical and molecular analysis of AMD comprises late AMD cases and controls recruited at Radboud University Medical Center, the Netherlands, and University of Cologne, Germany. All participants provided written informed consent for clinical examination, epidemiological data collection, and blood sampling for biochemical and genetic analyses. Serum samples were used for FHR-4/FH measurements.
Ethics oversight	Donor eye tissue was obtained from Manchester Eye Tissue Repository (ethically approved Research Tissue Bank, UK NHS Health Research Authority ref 15/NW/0932). The banked tissue was collected and stored within 48 hours of death; there was prior informed consent for research use. Human Tissue Act 2004 (UK) guidelines were followed. For all studies, ethical approval was obtained from either national or local ethics committees and adhered to the tenets of the Declaration of Helsinki.

Note that full information on the approval of the study protocol must also be provided in the manuscript.
